# Propane-1,3-diaminium pyridine-2,5-di­carboxyl­ate dimethyl sulfoxide mono­solvate

**DOI:** 10.1107/S1600536811004545

**Published:** 2011-02-12

**Authors:** Hossein Aghabozorg, Minoo Bayan, Masoud Mirzaei, Behrouz Notash

**Affiliations:** a Faculty of Chemistry, Islamic Azad University, North Tehran Branch, Tehran, Iran; b Department of Chemistry, School of Sciences, Ferdowsi University of Mashhad, Mashhad 917791436, Iran; c Department of Chemistry, Shahid Beheshti University, G. C., Evin, Tehran, 1983963113, Iran

## Abstract

In the crystal structure of the title solvated molecular salt, C_3_H_12_N_2_^2+^·C_7_H_3_NO_4_^2−^·C_2_H_6_OS, two amine groups of propane-1,3-diamine (pda) are protonated and two carb­oxy­lic acid groups of pyridine-2,5-dicarb­oxy­lic acid (2,5-pydcH_2_) are deprotonated. The crystal packing features N—H⋯O hydrogen bonds and weak C—H⋯O inter­molecular inter­actions.

## Related literature

Pyridine-2,5-dicarb­oxy­lic acid (2,5-pydcH_2_) can coordinate to metal centers (Pasdar *et al.*, 2011 ▶) or form hydrogen-bonded networks (Zeng *et al.*, 2005 ▶). For work by our group on the synthesis of proton-transfer compounds containing different proton donor and acceptor groups, see: Eshtiagh-Hosseini *et al.* (2010*a* ▶,*b* ▶); Aghabozorg *et al.* (2008 ▶, 2011 ▶). 
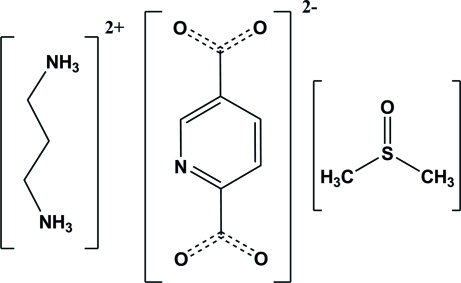


## Experimental

### 

#### Crystal data

C_3_H_12_N_2_^2+^·C_7_H_3_NO_4_^2−^·C_2_H_6_OS
*M* *_r_* = 319.39
Monoclinic, 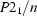

*a* = 11.984 (2) Å
*b* = 10.346 (2) Å
*c* = 12.942 (3) Å
β = 111.63 (3)°
*V* = 1491.6 (6) Å^3^
*Z* = 4
Mo *K*α radiation
μ = 0.24 mm^−1^
*T* = 120 K
0.4 × 0.3 × 0.3 mm

#### Data collection

STOE IPDS 2T diffractometer
12249 measured reflections
4010 independent reflections
3380 reflections with *I* > 2σ(*I*)
*R*_int_ = 0.035

#### Refinement

*R*[*F* ^2^ > 2σ(*F* ^2^)] = 0.041
*wR*(*F* ^2^) = 0.095
*S* = 1.07
4010 reflections
216 parameters
H atoms treated by a mixture of independent and constrained refinement
Δρ_max_ = 0.43 e Å^−3^
Δρ_min_ = −0.34 e Å^−3^

### 

Data collection: *X-AREA* (Stoe & Cie, 2005 ▶); cell refinement: *X-AREA*; data reduction: *X-AREA*; program(s) used to solve structure: *SHELXS97* (Sheldrick, 2008 ▶); program(s) used to refine structure: *SHELXL97* (Sheldrick, 2008 ▶); molecular graphics: *ORTEP-3 for Windows* (Farrugia, 1997 ▶); software used to prepare material for publication: *WinGX* (Farrugia, 1999 ▶).

## Supplementary Material

Crystal structure: contains datablocks I, global. DOI: 10.1107/S1600536811004545/jj2074sup1.cif

Structure factors: contains datablocks I. DOI: 10.1107/S1600536811004545/jj2074Isup2.hkl

Additional supplementary materials:  crystallographic information; 3D view; checkCIF report

## Figures and Tables

**Table 1:** Hydrogen-bond geometry (Å, °)

*D*—H⋯*A*	*D*—H	H⋯*A*	*D*⋯*A*	*D*—H⋯*A*
N2—H2*A*⋯O4^i^	0.91 (2)	1.96 (2)	2.8260 (16)	157.8 (17)
N2—H2*B*⋯O3^ii^	0.86 (2)	2.06 (2)	2.8461 (17)	151.0 (18)
N2—H2*C*⋯O2^iii^	0.92 (2)	1.84 (2)	2.7385 (17)	164.4 (18)
N3—H3*A*⋯O1^iv^	0.91 (2)	1.85 (2)	2.7369 (17)	161.6 (18)
N3—H3*B*⋯O3	0.890 (19)	2.073 (19)	2.8427 (16)	144.2 (16)
N3—H3*C*⋯O4^v^	0.86 (2)	1.96 (2)	2.7925 (17)	164.2 (18)
C8—H8*A*⋯O5^vi^	0.97	2.50	3.4614 (19)	170
C10—H10*A*⋯O5	0.97	2.53	3.4718 (19)	165
C11—H11*B*⋯O1^ii^	0.96	2.46	3.424 (2)	178

Symmetry codes: (i) 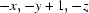; (ii) 
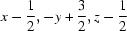
; (iii) 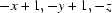; (iv) 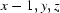; (v) 
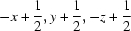
; (vi) 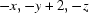.

**Fig. 1.**
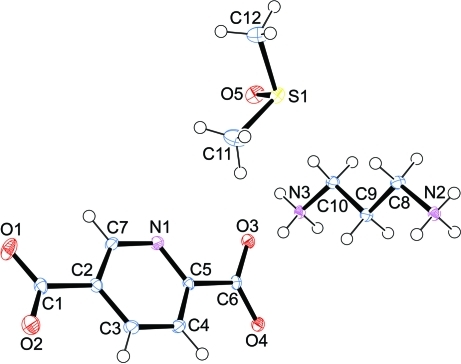
The molecular structure of title compound with displacement ellipsoids drawn at 50% probability level.

**Fig. 2.**
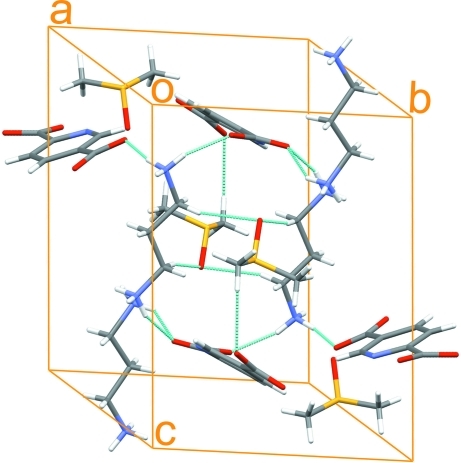
The packing diagram of the title compound, viewed down the a axis, showing N—H···O hydrogen bonds and weak C—H···O intermolecular interactions (dashed lines).

## supplementary crystallographic information

### Comment

Pyridine-2,5-dicarboxylic acid (2,5-pydcH_2_) can coordinate to metal centers (Pasdar *et al.*, 2011) or form hydrogen-bonded networks (Zeng *et al.*, 2005).  Our research group has been focused on synthesis of proton transfer compounds containing different proton donor and acceptor groups (Eshtiagh-Hosseini *et al.*, 2010*a*; Eshtiagh-Hosseini *et al.*, 2010*b*; Aghabozorg *et al.*, 2008, 2011).

We report here the synthesis and crystal structure of the title proton transfer compound, [pdaH2]^2+^.[2,5-pydc]^2-^.(DMSO). The asymmetric unit contains deprotonated pyridine-2,5-dicarboxylic acid, diprotonated propane-1,3-diamine, and one DMSO solvent molecule (Fig. 1). Crystal packing is stabilized by N—H···O hydrogen bonds and weak C—H···O intermolecular interactions (Fig. 2 & Table 1).

### Experimental

Propane-1,3-diamine (0.07 g, 0.29 ml, 1 mmol) was added to a DMSO/H_2_O solution of pyridine-2,5-dicarboxylic acid (0.17 g, 1 mmol) (13 ml) at room temperature. The suitable crystals for X-ray diffraction experiment were isolated by slow evaporation of the solvent after two months.

### Refinement

Nitrogen-bound H atoms were found in difference Fourier map and refined isotropically without restraint. Carbon-bound H atoms were positioned geometrically and refined as riding atoms with C—H distances of 0.93 Å (aromatic) and 0.97 Å (CH_2_) and were refined with *U*iso(H) = 1.2 *Ueq*(C).

### Crystal data

**Table:**

C_3_H_12_N_2_^2+^·C_7_H_3_NO_4_^2^^−^·C_2_H_6_OS	*F*(000) = 680
*M**_r_* = 319.39	*D*_x_ = 1.422 Mg m^−^^3^
Monoclinic, *P*2_1_/*n*	Mo *K*α radiation, λ = 0.71073 Å
Hall symbol: -P 2yn	Cell parameters from 4010 reflections
*a* = 11.984 (2) Å	θ = 2.6–29.2°
*b* = 10.346 (2) Å	µ = 0.24 mm^−^^1^
*c* = 12.942 (3) Å	*T* = 120 K
β = 111.63 (3)°	Block, colorless
*V* = 1491.6 (6) Å^3^	0.4 × 0.3 × 0.3 mm
*Z* = 4	

### Data collection

**Table:**

STOE IPDS 2T diffractometer	3380 reflections with *I* > 2σ(*I*)
Radiation source: fine-focus sealed tube	*R*_int_ = 0.035
graphite	θ_max_ = 29.2°, θ_min_ = 2.6°
Detector resolution: 0.15 pixels mm^-1^	*h* = −16→14
rotation method scans	*k* = −14→14
12249 measured reflections	*l* = −17→17
4010 independent reflections	

### Refinement

**Table:**

Refinement on *F*^2^	Primary atom site location: structure-invariant direct methods
Least-squares matrix: full	Secondary atom site location: difference Fourier map
*R*[*F*^2^ > 2σ(*F*^2^)] = 0.041	Hydrogen site location: inferred from neighbouring sites
*wR*(*F*^2^) = 0.095	H atoms treated by a mixture of independent and constrained refinement
*S* = 1.07	*w* = 1/[σ^2^(*F*_o_^2^) + (0.0447*P*)^2^ + 0.6814*P*] where *P* = (*F*_o_^2^ + 2*F*_c_^2^)/3
4010 reflections	(Δ/σ)_max_ < 0.001
216 parameters	Δρ_max_ = 0.43 e Å^−^^3^
0 restraints	Δρ_min_ = −0.34 e Å^−^^3^

### Special details

**Table:**

Geometry. All e.s.d.'s (except the e.s.d. in the dihedral angle between two l.s. planes) are estimated using the full covariance matrix. The cell e.s.d.'s are taken into account individually in the estimation of e.s.d.'s in distances, angles and torsion angles; correlations between e.s.d.'s in cell parameters are only used when they are defined by crystal symmetry. An approximate (isotropic) treatment of cell e.s.d.'s is used for estimating e.s.d.'s involving l.s. planes.
Refinement. Refinement of *F*^2^ against ALL reflections. The weighted *R*-factor *wR* and goodness of fit *S* are based on *F*^2^, conventional *R*-factors *R* are based on *F*, with *F* set to zero for negative *F*^2^. The threshold expression of *F*^2^ > σ(*F*^2^) is used only for calculating *R*-factors(gt) *etc*. and is not relevant to the choice of reflections for refinement. *R*-factors based on *F*^2^ are statistically about twice as large as those based on *F*, and *R*- factors based on ALL data will be even larger.

### Fractional atomic coordinates and isotropic or equivalent isotropic displacement parameters (Å^2^)

**Table:**

	*x*	*y*	*z*	*U*_iso_*/*U*_eq_	
S1	0.25330 (3)	0.98812 (3)	0.01042 (3)	0.01677 (10)	
O1	0.89632 (10)	0.63514 (12)	0.26504 (10)	0.0237 (2)	
O2	0.87767 (10)	0.45057 (12)	0.17030 (11)	0.0271 (3)	
O3	0.28902 (9)	0.63320 (10)	0.16349 (8)	0.0147 (2)	
O4	0.28589 (9)	0.41856 (10)	0.13819 (8)	0.0145 (2)	
O5	0.26031 (11)	1.00924 (11)	0.12737 (9)	0.0216 (2)	
N1	0.52969 (11)	0.63725 (11)	0.20599 (10)	0.0136 (2)	
N2	−0.08584 (11)	0.67039 (12)	−0.18378 (10)	0.0120 (2)	
H2A	−0.1484 (18)	0.6233 (18)	−0.1797 (15)	0.018 (5)*	
H2B	−0.1114 (17)	0.7160 (19)	−0.2439 (16)	0.019 (5)*	
H2C	−0.0253 (19)	0.619 (2)	−0.1876 (16)	0.024 (5)*	
N3	0.09589 (11)	0.71239 (12)	0.22640 (10)	0.0122 (2)	
H3A	0.0361 (18)	0.6692 (19)	0.2400 (15)	0.020 (5)*	
H3B	0.1526 (17)	0.6583 (18)	0.2240 (14)	0.013 (4)*	
H3C	0.1289 (18)	0.768 (2)	0.2777 (16)	0.021 (5)*	
C1	0.83737 (12)	0.54143 (14)	0.20910 (12)	0.0143 (3)	
C2	0.70396 (12)	0.53745 (13)	0.18810 (11)	0.0116 (2)	
C3	0.63536 (13)	0.43090 (13)	0.13750 (12)	0.0151 (3)	
H3	0.6694	0.3631	0.1122	0.018*	
C4	0.51512 (13)	0.42656 (13)	0.12501 (12)	0.0144 (3)	
H4	0.4682	0.3550	0.0929	0.017*	
C5	0.46609 (11)	0.53089 (13)	0.16125 (11)	0.0108 (2)	
C6	0.33590 (12)	0.52826 (13)	0.15352 (10)	0.0111 (2)	
C7	0.64639 (12)	0.63845 (13)	0.21994 (11)	0.0133 (3)	
H7	0.6915	0.7109	0.2528	0.016*	
C8	−0.03989 (13)	0.75898 (13)	−0.08677 (11)	0.0144 (3)	
H8A	−0.1022	0.8202	−0.0896	0.017*	
H8B	0.0278	0.8073	−0.0907	0.017*	
C9	−0.00090 (12)	0.68569 (13)	0.02247 (11)	0.0134 (3)	
H9A	0.0622	0.6249	0.0264	0.016*	
H9B	−0.0682	0.6374	0.0272	0.016*	
C10	0.04461 (13)	0.78090 (13)	0.11828 (11)	0.0139 (3)	
H10A	0.1056	0.8357	0.1084	0.017*	
H10B	−0.0210	0.8357	0.1183	0.017*	
C11	0.37246 (17)	0.88029 (16)	0.02034 (14)	0.0256 (3)	
H11A	0.4460	0.9123	0.0744	0.038*	
H11B	0.3803	0.8739	−0.0507	0.038*	
H11C	0.3553	0.7965	0.0427	0.038*	
C12	0.31441 (17)	1.13018 (16)	−0.02737 (15)	0.0264 (3)	
H12A	0.2650	1.2033	−0.0275	0.040*	
H12B	0.3167	1.1191	−0.1002	0.040*	
H12C	0.3943	1.1444	0.0253	0.040*	

### Atomic displacement parameters (Å^2^)

**Table:**

	*U*^11^	*U*^22^	*U*^33^	*U*^12^	*U*^13^	*U*^23^
S1	0.01593 (17)	0.01708 (17)	0.01747 (17)	−0.00372 (13)	0.00635 (13)	−0.00230 (13)
O1	0.0141 (5)	0.0294 (6)	0.0311 (6)	−0.0076 (5)	0.0125 (5)	−0.0101 (5)
O2	0.0145 (5)	0.0236 (6)	0.0466 (7)	0.0026 (5)	0.0151 (5)	−0.0072 (5)
O3	0.0106 (4)	0.0155 (5)	0.0186 (5)	0.0014 (4)	0.0061 (4)	−0.0011 (4)
O4	0.0103 (4)	0.0146 (5)	0.0187 (5)	−0.0015 (4)	0.0054 (4)	0.0018 (4)
O5	0.0245 (6)	0.0222 (5)	0.0223 (5)	−0.0018 (4)	0.0137 (4)	−0.0039 (4)
N1	0.0118 (5)	0.0126 (5)	0.0174 (5)	0.0001 (4)	0.0065 (4)	−0.0009 (4)
N2	0.0098 (5)	0.0133 (5)	0.0133 (5)	−0.0003 (4)	0.0046 (4)	−0.0006 (4)
N3	0.0092 (5)	0.0134 (5)	0.0133 (5)	−0.0004 (5)	0.0035 (4)	0.0003 (4)
C1	0.0103 (6)	0.0176 (6)	0.0164 (6)	0.0004 (5)	0.0064 (5)	0.0038 (5)
C2	0.0099 (6)	0.0131 (6)	0.0131 (6)	0.0005 (5)	0.0057 (5)	0.0017 (5)
C3	0.0139 (6)	0.0126 (6)	0.0208 (6)	0.0015 (5)	0.0087 (5)	−0.0021 (5)
C4	0.0122 (6)	0.0118 (6)	0.0195 (6)	−0.0013 (5)	0.0063 (5)	−0.0021 (5)
C5	0.0085 (6)	0.0121 (6)	0.0124 (5)	0.0006 (5)	0.0044 (5)	0.0022 (5)
C6	0.0082 (6)	0.0152 (6)	0.0101 (5)	0.0001 (5)	0.0035 (4)	0.0008 (5)
C7	0.0116 (6)	0.0114 (6)	0.0172 (6)	−0.0021 (5)	0.0056 (5)	−0.0021 (5)
C8	0.0166 (7)	0.0125 (6)	0.0144 (6)	−0.0006 (5)	0.0059 (5)	−0.0008 (5)
C9	0.0121 (6)	0.0132 (6)	0.0145 (6)	−0.0004 (5)	0.0047 (5)	0.0000 (5)
C10	0.0149 (6)	0.0127 (6)	0.0137 (6)	0.0001 (5)	0.0049 (5)	0.0007 (5)
C11	0.0380 (10)	0.0197 (7)	0.0261 (8)	0.0078 (7)	0.0201 (7)	0.0009 (6)
C12	0.0351 (9)	0.0171 (7)	0.0315 (8)	−0.0015 (7)	0.0175 (7)	0.0028 (6)

### Geometric parameters (Å, °)

**Table:**

S1—O5	1.5007 (12)	C3—C4	1.3904 (19)
S1—C11	1.7791 (17)	C3—H3	0.9300
S1—C12	1.7888 (17)	C4—C5	1.3905 (18)
O1—C1	1.2594 (18)	C4—H4	0.9300
O2—C1	1.2442 (19)	C5—C6	1.5265 (18)
O3—C6	1.2508 (17)	C7—H7	0.9300
O4—C6	1.2645 (17)	C8—C9	1.5182 (19)
N1—C5	1.3423 (17)	C8—H8A	0.9700
N1—C7	1.3427 (18)	C8—H8B	0.9700
N2—C8	1.4866 (18)	C9—C10	1.5189 (19)
N2—H2A	0.91 (2)	C9—H9A	0.9700
N2—H2B	0.86 (2)	C9—H9B	0.9700
N2—H2C	0.92 (2)	C10—H10A	0.9700
N3—C10	1.4847 (18)	C10—H10B	0.9700
N3—H3A	0.91 (2)	C11—H11A	0.9600
N3—H3B	0.890 (19)	C11—H11B	0.9600
N3—H3C	0.86 (2)	C11—H11C	0.9600
C1—C2	1.5204 (19)	C12—H12A	0.9600
C2—C3	1.3867 (19)	C12—H12B	0.9600
C2—C7	1.3955 (18)	C12—H12C	0.9600
			
O5—S1—C11	105.87 (8)	N1—C7—C2	123.87 (13)
O5—S1—C12	106.25 (7)	N1—C7—H7	118.1
C11—S1—C12	97.83 (8)	C2—C7—H7	118.1
C5—N1—C7	117.60 (12)	N2—C8—C9	111.69 (11)
C8—N2—H2A	109.7 (12)	N2—C8—H8A	109.3
C8—N2—H2B	108.8 (13)	C9—C8—H8A	109.3
H2A—N2—H2B	108.5 (17)	N2—C8—H8B	109.3
C8—N2—H2C	110.5 (12)	C9—C8—H8B	109.3
H2A—N2—H2C	112.2 (17)	H8A—C8—H8B	107.9
H2B—N2—H2C	107.1 (17)	C8—C9—C10	109.33 (12)
C10—N3—H3A	109.5 (12)	C8—C9—H9A	109.8
C10—N3—H3B	108.8 (11)	C10—C9—H9A	109.8
H3A—N3—H3B	111.2 (17)	C8—C9—H9B	109.8
C10—N3—H3C	108.6 (13)	C10—C9—H9B	109.8
H3A—N3—H3C	110.5 (17)	H9A—C9—H9B	108.3
H3B—N3—H3C	108.1 (17)	N3—C10—C9	111.05 (11)
O2—C1—O1	126.50 (14)	N3—C10—H10A	109.4
O2—C1—C2	116.52 (13)	C9—C10—H10A	109.4
O1—C1—C2	116.98 (13)	N3—C10—H10B	109.4
C3—C2—C7	117.59 (12)	C9—C10—H10B	109.4
C3—C2—C1	120.48 (12)	H10A—C10—H10B	108.0
C7—C2—C1	121.92 (12)	S1—C11—H11A	109.5
C2—C3—C4	119.29 (13)	S1—C11—H11B	109.5
C2—C3—H3	120.4	H11A—C11—H11B	109.5
C4—C3—H3	120.4	S1—C11—H11C	109.5
C3—C4—C5	118.97 (13)	H11A—C11—H11C	109.5
C3—C4—H4	120.5	H11B—C11—H11C	109.5
C5—C4—H4	120.5	S1—C12—H12A	109.5
N1—C5—C4	122.59 (12)	S1—C12—H12B	109.5
N1—C5—C6	116.55 (11)	H12A—C12—H12B	109.5
C4—C5—C6	120.85 (12)	S1—C12—H12C	109.5
O3—C6—O4	126.18 (12)	H12A—C12—H12C	109.5
O3—C6—C5	117.70 (12)	H12B—C12—H12C	109.5
O4—C6—C5	116.11 (12)		
			
O2—C1—C2—C3	6.0 (2)	C3—C4—C5—C6	−177.55 (12)
O1—C1—C2—C3	−173.18 (13)	N1—C5—C6—O3	16.67 (17)
O2—C1—C2—C7	−175.04 (14)	C4—C5—C6—O3	−164.35 (13)
O1—C1—C2—C7	5.8 (2)	N1—C5—C6—O4	−162.89 (12)
C7—C2—C3—C4	−2.7 (2)	C4—C5—C6—O4	16.09 (18)
C1—C2—C3—C4	176.36 (13)	C5—N1—C7—C2	1.7 (2)
C2—C3—C4—C5	1.6 (2)	C3—C2—C7—N1	1.1 (2)
C7—N1—C5—C4	−2.97 (19)	C1—C2—C7—N1	−177.95 (13)
C7—N1—C5—C6	175.99 (11)	N2—C8—C9—C10	−179.79 (11)
C3—C4—C5—N1	1.4 (2)	C8—C9—C10—N3	−173.86 (11)

### Hydrogen-bond geometry (Å, °)

**Table:**

*D*—H···*A*	*D*—H	H···*A*	*D*···*A*	*D*—H···*A*
N2—H2A···O4^i^	0.91 (2)	1.96 (2)	2.8260 (16)	157.8 (17)
N2—H2B···O3^ii^	0.86 (2)	2.06 (2)	2.8461 (17)	151.0 (18)
N2—H2C···O2^iii^	0.92 (2)	1.84 (2)	2.7385 (17)	164.4 (18)
N3—H3A···O1^iv^	0.91 (2)	1.85 (2)	2.7369 (17)	161.6 (18)
N3—H3B···O3	0.890 (19)	2.073 (19)	2.8427 (16)	144.2 (16)
N3—H3C···O4^v^	0.86 (2)	1.96 (2)	2.7925 (17)	164.2 (18)
C8—H8A···O5^vi^	0.97	2.50	3.4614 (19)	170
C10—H10A···O5	0.97	2.53	3.4718 (19)	165
C11—H11B···O1^ii^	0.96	2.46	3.424 (2)	178

Symmetry codes: (i) −*x*, −*y*+1, −*z*; (ii) *x*−1/2, −*y*+3/2, *z*−1/2; (iii) −*x*+1, −*y*+1, −*z*; (iv) *x*−1, *y*, *z*; (v) −*x*+1/2, *y*+1/2, −*z*+1/2; (vi) −*x*, −*y*+2, −*z*.
